# Metabolic syndrome and thyroid Cancer: risk, prognosis, and mechanism

**DOI:** 10.1007/s12672-022-00599-7

**Published:** 2023-02-22

**Authors:** Ling-Rui Li, Jun-Long Song, Han-Qing Liu, Chuang Chen

**Affiliations:** Department of Breast and Thyroid Surgery, Renmin Hospital of Wuhan University, No. 238 Jieang Road, Wuchang District, Wuhan, 430060 Hubei PR China

**Keywords:** Thyroid cancer, Metabolic syndrome, Risk factor, Prognosis, Biological mechanism

## Abstract

The increasing incidence of thyroid cancer (TC) cannot be fully explained by overdiagnosis. Metabolic syndrome (Met S) is highly prevalent due to the modern lifestyle, which can lead to the development of tumors. This review expounds on the relationship between Met S and TC risk, prognosis and its possible biological mechanism. Met S and its components were associated with an increased risk and aggressiveness of TC, and there were gender differences in most studies. Abnormal metabolism places the body in a state of chronic inflammation for a long time, and thyroid-stimulating hormones may initiate tumorigenesis. Insulin resistance has a central role assisted by adipokines, angiotensin II, and estrogen. Together, these factors contribute to the progression of TC. Therefore, direct predictors of metabolic disorders (e.g., central obesity, insulin resistance and apolipoprotein levels) are expected to become new markers for diagnosis and prognosis. cAMP, insulin-like growth factor axis, angiotensin II, and AMPK-related signaling pathways could provide new targets for TC treatment.

## Highlights

- Met S and its components were associated with an increased risk and aggressiveness of thyroid cancer.
- The obese males have a tighter association with thyroid cancer, and females have a higher thyroid cancer risk among diabetes.
- Thyroid-stimulating hormones may be a switch for thyroid carcinogenesis.
- Insulin resistance plays a critical role in the development of thyroid cancer.
- Abnormal levels of adipocytokines and blood pressure related regulatory hormone assist in accelerating thyroid cancer progression.

## Introduction

The incidence of thyroid cancer (TC) has been on the rise in recent years. Most cases involve small papillary thyroid cancer (PTC) with a good prognosis [1, 2]. Some scholars believe that the use of ultrasound and fine-needle biopsy technologies enables the early detection and treatment of subclinical TC [3]. However, PTC with large sizes [4] and advanced stages [2] have also increased at diagnosis. The expert consensus in China points out that the rising numbers of TC patients not only result from the early screening, but may also be related to factors such as hormones and obesity [5]. Thus, other modifiable factors, such as lifestyle and environment, cannot be ignored. Metabolic syndrome (Met S), a group of reversible and preventable disorders including central obesity, diabetes, hypertension, and dyslipidemia, has become a global problem due to the changes in people’s eating and leisure habits. Up to 15% of the people in China have metabolic disturbances [6]. In Western countries, the incidence rate is 20%- 25% among adults, and it increases with age reaching over 40% in those older than 60 years [7]. Met S itself changes the organism’s microenvironment and is accompanied by insulin resistance, both of which are related to tumor formation. Thus, we reviewed the relationship between Met S and TC risk and prognosis, and its possible biological mechanism.

## **Metabolic syndrome, its components, and thyroid Cancer risk**

### **Metabolic syndrome and thyroid cancer risk**

Met S has been said to possibly increase the risk of disease, including carcinomas. Studies have demonstrated that Met S increases the risk of various carcinogenesis apparatuses including the liver, colorectum, and mammary tissue [8], but the association between TC and Met S is inconsistent. In a prospective study by Almquist et al. [9], they investigated the possible impacts of all five Met S components on TC, but no significant association was observed using z-score calculation.  A recent large study found that Met S is a risk factor for TC after adjusting for age, sex, smoking status, alcohol consumption, physical activity, income, and affliction with chronic kidney disease. The risk of TC is related to the number of aberrant metabolic components [10]. In agreement with the results of this study, López-Jiménez then showed that the risk of TC was 1.29, 1.57, and 1.71 times, respectively, in participants with one, two, and three or more abnormal metabolic factors than in the metabolically healthy population [11]. However, Nguyen et al. [12] further showed that Women with Met S are at increased TC risk regardless of normal weight or obesity (BMI≥25 kg/m ), but this positive correlation was not found in men.

### Obesity and thyroid cancer risk

Body mass index (BMI) is the most commonly used index to describe the degree of human obesity, and it is also the most frequent measure used to evaluate obesity and TC risk. Based on the World Health Organization (WHO) definition of obesity in Asians, Son et al. [13] concluded that obesity was positively correlated with the risk of TC after an average follow-up of 7 years in Koreans aged over 20 years. A meta-analysis including 22 prospective studies showed that the risk of TC increased by 6%, 13%, and 7%, respectively, for every 5 kg/m^2^ increase in total BMI, youth BMI, and middle-aged and elderly BMI [14]. The results obtained by Kwon et al. [15] are consistent with those of this meta-analysis and showed that weight gain significantly increased the incidence of TC (hazard ratio [HR] = 1.15, 95% confidence interval [CI] 1.11–1.19), but weight loss was a protective factor for TC (HR = 0.89, 95% CI 0.86–0.93). While evaluating different TC histological subtypes, many studies found that obesity was positively correlated with the risk of PTC, follicular TC (FTC), and undifferentiated TC. However, obesity had a surprisingly negative correlation with the risk of medullary TC (MTC) (relative risk = 0.50, 95% CI:0.27–0.92) [16]. This reminds us of the differences in the origin and function of tumor cells. The first three types originate from thyroid epithelial cells, which secrete thyroid hormones and participate in the regulation of human metabolism. However, MTC comes from parafollicular C cells, which secrete calcitonin to maintain calcium balance. This may suggest the importance of thyroid hormones in metabolic disorders and tumorigenesis.

Most studies indicated that obesity significantly increased the risk of TC in male patients [13, 14, 17], whereas the correlation was stronger in female patients with BRAF-mutated PTC [18]. BMI, as a systemic index, reflects the amount of body fat. But the distribution of fat, especially that in the abdominal viscera, can better predict metabolic complications and neoplasia. Moreover, body composition changes with age; postmenopausal women experience shortened height, decreased skeletal muscle, and increased central fat, for example. And patient’s self-reported values were used to calculate their BMI in some studies. However, men tend to overestimate their height, and women are more likely to underestimate their weight [19]. The limited accuracy of BMI may explain why those studies failed to reveal the relationship or the gender difference between BMI and TC risk. [20, 21]. The new diagnostic criteria for Met S further highlights the importance of central obesity. However, predicting the risk of TC is still controversial by using the waist, neck, or hip circumference or waist-hip ratio. Several studies showed that TC risk increased with the augment in the waist or hip circumferences and the waist-hip ratio [16, 22]. Kitahara et al. [23] found that after following nearly 200,000 people for an average of 10 years, the TC risk in males with abdominal obesity was higher than that in normal BMI, overweight, and obesity groups. Recalde et al. [20] suggested that in 19 kinds of cancer, changes (1 standard deviation increase) in BMI and waist circumference showed significant cancer risk consistency, whereas this increase failed to sign an increased TC risk.

Obesity is generally associated with an increased risk of TC, particularly in differentiated TC (DTC) and male patients. Weight control, especially in young adulthood, may reduce the risk of TC. However, the predictive value of different obesity indices needs to be further studied for TC risk.

### Hyperglycemia and thyroid cancer risk

According to the latest results from the International Diabetes Federation Diabetes Atlas, about 463 million people (9.3%) live with diabetes in 2019 and this number has been continuously increasing up to 700 million (10.9%) in 2045. Simultaneously, about 7.5% of the global population has impaired glucose tolerance, and the figure is expected to 8.6% in 2045 [24]. High fasting blood glucose (≥ 6.1 mmol/L) or diabetes mellitus suggests insulin resistance but its impact on TC risk is still controversial. A matched study showed that the history and duration of diabetes were not associated with TC risk , but women with diabetes fewer than 5 years have a reduced risk of TC [25]. However, up to 28% of Chinese cancer patients suffered from diabetes or impaired fasting glucose, both of which are associated with an increased risk of TC [26]. Lo et al. [27] analyzed the relationship between type 2 diabetes and cancer risk and found that diabetes was a risk factor for TC. Li et al. [28] included 16 cohort studies in their meta-analysis and confirmed this positive correlation. Subgroup analysis revealed that diabetes increased the risk of TC by 11% in females, but no significant TC risk was found in males with diabetes [28]. The gender disparity in risk could also be observed in another meta-analysis [29]. This relationship may vary from country to country, age, and course of diabetes [27].

Several studies have shown that drugs for diabetes at early stage have anti-tumor properties such as metformin and acarbose [30, 31]. This may partly explain the declined risk of TC in women with short-course diabetes.

Insulin resistance is an important feature of type 2 diabetes. About 56% of PTC patients and 25% of FTC patients have insulin resistance. Moreover, insulin resistance was five times more common in DTC patients than in euthyroid individuals [32]. Directly assessing the TC risk posed by insulin resistance level may be more clinically significant. Heidar et al. [33] confirmed that the homeostatic model assessment of the insulin resistance model (HOMA-IR) was associated with TC occurrence (Odds Ratio [OR] = 2.43, 95% CI 1.35–5.51). A meta-analysis showed that TC risk was 1.40 times and 1.59 times higher in patients with abnormal blood glucose and insulin resistance, respectively [17]. Bae et al. [34] excluded the effects of drugs and their results showed that elevated insulin, blood glucose level and HOMA-IR score were risk factors for TC females. Therefore, most studies suggest that hyperglycemia increases the risk of TC, especially in women, but several factors need to be further considered like age, insulin resistance, duration of diabetes, and antidiabetic agents.

### Hypertension and thyroid Cancer risk 

Given the current prevalence of TC and hypertension, it is crucial to explore the relationship between these two diseases. In 2018, a meta-analysis first confirmed the link between hypertension and TC, showing a 14% increased risk of TC in hypertensive individuals [17]. Park et al. [10] showed that this positive correlation existed in both the obese (OR = 1.03, 95% CI 1.01–1.06) and the non-obese (OR = 1.08, 95% CI 1.05–1.11).

Patients who have been diagnosed with high blood pressure take antihypertension drugs for life, a much longer time than that of the initial trial to test drug safety. It is worth mentioning that there are two opposing views on the impact of antihypertensive drugs on cancer: one is that the long-term use of certain antihypertensive drugs increases the risk of carcinoma. A multivariate analysis showed that ovarian cancer patients, who took calcium channel blockers before surgery, had an increased risk of death compared with those who did not take a such drug (HR: 1.49, 95% CI 1.13–1.96). However, no association was found between taking thiazide diuretics, beta-blockers, or angiotensin-converting enzyme inhibitors (ACEI) and cancer risk [35]. A meta-analysis found similar results from 33 studies [36]. Of the four most commonly used antihypertensive medicines, only calcium channel blockers were found to be weakly correlated with cancer risk (HR: 1.06, 95% CI 1.01–1.11).

The opposite views hold that antihypertensive drugs have no influence on cancer occurrence or even improve cancer prognosis. In a meta-analysis, Cancer patients had longer survival using AECI or angiotensin II (Ang II) receptor blocker (ARB) [37]. Biological mechanism of antihypertensive drugs may vary greatly on cancer risk and progression. A study on Swiss men with high systolic blood pressure (SBP) showed that the men had a 41% higher risk of malignant tumors after an adjustment for different agents [38]. Unfortunately, the study did not involve TC patients, but its results may suggest that SBP plays a larger role in tumorigenesis induced by hypertension.

### Dyslipidemia and thyroid cancer risk

According to Met S diagnostic criteria, dyslipidemia is regarded as an increase in serum triglyceride (TG) or a decrease in high-density lipoprotein cholesterol (HDL-C). In China, over 40% of adults have dyslipidemia [39]. The burden of dyslipidemia and its complications is continually worsening. The blood lipid profile of Chinese TC female patients showed higher TG and lower HDL-C levels compared to those with benign tumors (P < 0.05) [40]. Park et al. [10] showed that low HDL-C is an unfavorable factor for TC, whereas high TG is a protective factor. In a prospective study on cancer risk in Austria, the investigators enrolled 101 patients with TC and found that a high TG level was associated with an increased risk of TC (HR = 1.96, 95%CI 1.00–3.84) [41]. However, a recent meta-analysis of 42 studies showed no association between the risk of TC and dyslipidemia (neither high TG level [OR = 1.01, 95%CI 0.91–1.12] nor high total cholesterol level [OR = 1.09, 95%CI 0.98–1.21]) [17].

Lipid physiological functions may lead to different tumorigenesis risks. HDL-C transports cholesterol from peripheral tissues to the liver for metabolism, limiting cholesterol deposition in the blood vessels and thereby reducing vascular endothelial damage. A rise in the TG level often reflects an increase in very low-density lipoprotein and its residual particles. These smaller particles cause direct damage to the blood vessels, and vascular injury accelerates cell invasion and metastasis. Fibrates are often used for the lipid-lowering treatment of patients with high TG levels, and may have protective functions in tumor progression in the breast, gastrointestinal tract, respiratory tract and urinary system [42, 43]. However, the aforementioned studies lacked data on whether the patients took drugs, if they did, what drugs they took, and how they took these.

## Metabolic syndrome, its components, and thyroid Cancer prognosis

### Metabolic syndrome and thyroid cancer prognosis

Met S is a risk factor for the postoperative recurrence of breast cancer, prostate cancer, colorectal cancer, and other malignant tumors [44–46]. Our previous study first showed that PTC with Met S was related to tumor size  > 1 cm, lymph node metastasis, and later TNM stage [47]. Aggressive clinicopathological features are prognostic factors for TC, but major studies, including ours, lacked the long-term follow-up to verify the effect of Met S on TC survival .

### Obesity and thyroid cancer prognosis

Many studies have investigated the relationship between BMI and TC prognosis. In a large retrospective analysis by Li et al. [48], obesity was correlated with PTC diameter  > 1 cm, multifocal carcinoma, extrathyroidal extension (ETE), lymph node metastasis and metastatic number according to the WHO criteria or the BMI classification in China. A meta-analysis yielded similar results: PTC patients had an increased risk of invasive pathological features with a BMI increase [49]. TNM staging is clinically used to evaluate patients’ prognosis, Warakomski et al. [50] found that abdominal adiposity was a risk factor for TNM stage III-IV, while gender was adjusted only in the multivariate analysis. But most studies including ours failed to show that obesity is associated with later TNM stages [47, 49, 51]. Tumorigenesis is a long process of metabolic disorders where different components may be responsible for specific stages. The clinical role of obesity, especially central adiposity, needs to be confirmed after controlling the “interfering effect” in large samples.

Wu et al. [52] conducted a matched retrospective analysis of 57 patients with recurrent TC and found that obesity was an independent risk factor for recurrence. Patients with normal weights had higher disease-free survival rates than those with underweight, overweight, or obesity. However, in the study by Kim et al. [51], 43 of the 2057 PTC patients had recurrences after a 7-year follow-up, and there was no statistically significant difference in recurrence between the obese group and the normal BMI group. Feng et al. [53] also did not find a relationship between obesity and local recurrence of PTC after median follow-up of 2.4 years in 417 PTC patients. Well differentiated PTC has a favorable prognosis, specifically its 10-year survival rate is more than 90%. Therefore, long-term follow-up is still needed to prove the relationship between obesity and clinical prognosis of TC.

### Hyperglycemia and thyroid cancer prognosis

In a study by Hu et al. [54], fasting blood glucose level increased the risk of PTC diameter  > 1 cm. Mele et al. [55] followed 57 DTC patients who underwent total thyroidectomy for 3 years, and found that their insulin levels and HOMA-IR scores were associated with the recurrence of TC (p < 0.05). However, neither Chen et al. [56] nor Song et al. [47] demonstrated a relationship between hyperglycemia and the clinicopathological features of TC. This may be because a large proportion of the patients in the such study reported a history of diabetes by themselves. In a study involving 1687 DTC patients, 122 of whom also had type 2 diabetes, their 5-, 10- and 20-year specific survival rates were 82.2%, 72.9%, and 36.5% respectively in the type 2 diabetes group and 94.9%, 91.4%, and 61.3% in the non-diabetes group. Multivariate analysis showed that type 2 diabetes was an independent risk factor for TC-specific death [56].

### Hypertension and thyroid cancer prognosis

To date, no large-scale study has explored the relationship between hypertension and TC prognosis. In a cancer project, cancer mortality increased in both men (OR = 1.12, 95% CI 1.08–1.15) and women (OR = 1.06, 95% CI 1.02–1.11) with every 10mmHg rise in mean arterial pressure [57]. However, this project only included a small amount of TC patients. The results of our previous study showed that hypertension was related to PTC diameter > 1 cm and TNM stages II-III [47].

### Dyslipidemia and thyroid cancer prognosis

Li et al. [58] first analyzed the relationship between blood lipid levels and the clinicopathological features of PTC and found that a high TG level was a risk factor for ETE and distant metastasis in female PTC patients. Specifically, the risk of ETE increased by 1.35 times when the TG level rose from 0.90 mmol/L to1.93 mmol/L. Nevertheless, our study did not present the relationships between high TG level and tumor size, multifocal tumor, lymph node metastasis, and TNM stage in PTC patients [47]. In terms of HDL-C, some researchers observed higher levels in patients with PTC lymph node metastasis than in those without metastasis (P = 0.024), and there was no statistically significant difference in FTC and MTC [40]. However, Revilla et al. [59] indicated that the HDL-C levels in the invasive TC group (high-risk PTC, dedifferentiated/undifferentiated TC) were similar to those in the benign tumor group. Our multivariate analysis showed that a low HDL-C level was a risk factor for tumor size > 1 cm and lymph node metastasis [47]. A meta-analysis of cancer survivors showed that patients with high HDL-C levels before a diagnosis of malignancy had a 37% lower risk of death and 35% lower risk of recurrence than those with low HDL-C levels [60].

In previous studies, there was a controversy over the relationship between dyslipidemia and TC prognosis. HDL-C, a potential tumor inhibitor, plays anti-inflammatory and antioxidant roles mainly through apolipoprotein A1(ApoA1) and paraoxonase 1(PON1). Studies have shown that a preoperative decrease in ApoA1 level is related to aggressive tumor features and shortened disease-free survival in solid tumors [61]. Therefore, the important structural fragment of HDL-C may be a guiding predictor for PTC patients with abnormal HDL-C metabolism.

Most studies held that Met S is related to the occurrence of TC, although this relationship is not conclusive (Table 1). Future research should explore more diagnostic indicators in depth after adjusting for age, gender, treatment time, and methods. The effects of Met S components differ by gender on TC. Obese males have an increased TC risk, while women had a higher risk in patients with hyperglycemia.

**Table 1:** Relationship between metabolic disorders and thyroid cancer risk

Author	Year	Study design	Metabolic status	Total patients	TC patients	Measurement	95%CI
Almquist et al.[9]	2011	PCS	Met S score	578,700	388	RR _males_=1.13	0.94–1.35
						RR _females_=1.00	0.87–1.15
Park et al.[10]	2020	RCS	Met S	9,890,917	77,133	HR = 1.15	1.13–1.17
			Hypertension			HR = 1.08	1.06–1.10
			HDL-C			HR = 1.17	1.15–1.19
			Triglycerides			HR = 0.96	0.95–0.98
López-Jiménez et al.[11]	2022	RCS	Met S	13,626	2,709	OR = 1.71	1.50–1.95
Nguyen et al.[12]	2022	PCS	Met S	160,650	471	HR _normal weight_ =1.57*	1.02–2.40
						HR _obesity_ =1.71*	1.21–2.41
Son et al.[13]	2018	RCS	Obesity	351,402	3,308	HR _BMI_ =1.27	1.05–1.55
Kitahara et al.[14]	2016	Meta analysis	Obesity	2,094,047	2,996	HR _BMI per 5 kg/m_ =1.06	1.02–1.10
						HR _WC per 5 cm_ =1.03	1.01–1.05
Kwon et al.[15]	2019	RCS	Obesity	11,323,006	50,464	HR _BMI_ =1.52	1.45–1.59
Schmid et al.[16]	2015	Meta analysis	Obesity	NA	12,199	RR = 1.55	1.21–1.99
Yin et al.^18^	2018	Meta analysis	Hyperglycemia	21,713	4,707	RR _insulin resistance_ =1.59	1.12–2.27
						RR _diabetes_ =1.40	1.15–1.70
			Hypertension			RR = 1.34	1.22–1.47
			Dyslipidemia			RR = 1.05	0.97–1.13
Rahman et al.[18]	2020	RCS	Obesity	2070	1013	OR = 1.72	1.37–2.16
Youssef et al.^22^	2020	Meta analysis	Obesity	24,489,477	18,015	RR = 1.5	1.45–1.55
Recalde et al.^21^	2021	PCS	Obesity	3, 658,417	2,688	RR _BMI per 1SD_ =1.08	0.94–1.26
						RR _WC per 1SD_ =1.15	0.98–1.34
Kitahara et al.^23^	2011	PCS	Obesity	197,710	210	HR _WC male_ =1.79	1.21–2.63
						HR _WC female_ =1.54	1.05–2.26
Kitahara et al.[23]	2020	PCS	Obesity	457,331	604	HR _BMI_ =1.3	1.05–1.62
Wang et al.[25]	2021	Case-control	Hyperglycemia	5874	2,937	OR _diabetes_ =0.75	0.21–2.73
Zhan et al.[26]	2009	RCS	Hyperglycemia	4,421	73	OR _diabetes_ =14.32	1.56-131.49
						OR _FPG_ =1.49	1.09–2.02
Lo et al.[27]	2012	RCS	Hyperglycemia	1,790,868	1,309	HR _diabetes_ =1.17	1.05–1.31
Li et al.[28]	2017	Meta analysis	Hyperglycemia	10,725,884	8,032	RR _diabetes_ =1.2	1.09–1.33
Yeo et al.[29]	2014	Meta analysis	Hyperglycemia	NA	3566	RR _diabetes_ =1.18	1.08–1.28
Heidar et al[33]	2017	Case-control	Hyperglycemia	60	30	OR _insulin resistance_=4.95	1.27–17.6
						OR _HOMA−IR_ =2.43	1.35–5.51
Bae et al.[34]	2015	RCS	Hyperglycemia	1,272	735	OR _insulin_=2.88	2.01–4.11
						OR _glucose_ = 9.32	2.81–5.89
						OR _HOMA−IR_ = 4.07	6.28–13.83
Ulmer et al.[41]	2009	PCS	Triglyceride	156,153	101	HR = 1.96	1.00-3.84

*PCS* prospective cohort study, *RCS* retrospective cohort study, *Met S* metabolic syndrome, *RR* risk ratio, *HR* hazard ratio, *OR* odd ratio, *HDL-C* high-density lipoprotein cholesterol, *NA* not available, *BMI* body mass index, *WC* waist circumference, *SD* standard deviation, *FSG* fasting serum glucose, *HOMA-IR* homeostatic model assessment for insulin resistance, *CI* confidence interval

*The population in test group are metabolically unhealthy women with normal weight or obesity

Similarly, it is not yet clear whether Met S affects the prognosis of TC. Most studies only explored the correlation between Met S and the clinicopathological features of TC patients, and their results showed that Met S is related to the aggressiveness of TC (Table 2). Further confirmation of the prognostic value of central obesity, insulin resistance, ApoA1, and PON1 must be obtained in large sample size and after long-term follow-up .

**Table 2:** Relationship between metabolic disorders and thyroid cancer prognosis

Author	Year	Study design	TC patients	Metabolic status	Outcome	Measurement	95%CI
Song et al.[47]	2021	RCS	745	Met S	tumor size > 1 cm	OR = 2.29	1.31–4.03
					LNM	OR = 1.97	1.11–3.51
					TNM stage	OR = 7.92	1.59–39.34
				Diabetes	tumor size > 1 cm	OR = 1.53	0.86–2.72
					LNM	OR = 1.48	0.82–2.70
					multifocality	OR = 1.22	0.66–2.25
					TNM stage	OR = 1.15	0.38–3.49
				Blood pressured ≥ 130/85mmHg	tumor size > 1 cm	OR = 1.65	1.17–2.32
					TNM stage	OR = 3.99	1.56–10.21
				High TG	tumor size > 1 cm	OR = 1.12	0.75–1.67
					LNM	OR = 0.79	0.52–1.20
					multifocality	OR = 1.37	0.90–2.08
					TNM stage	OR = 1.11	0.48–2.55
Li et al.[48]	2020	RCS	13,995	Obesity	tumor size size	OR _BMI_ =1.74	1.49–2.04
					multifocality	OR _BMI_ =1.48	1.29–1.71
					ETE	OR _BMI_ =1.37	1.18–1.60
					LNM	OR _BMI_ =1.58	1.08–2.31
					number of LNM	OR = 1.49	1.30–1.72
O’Neill et al.[49]	2021	Meta analysis	35,237	Obesity	tumor size size	OR _BMI_ =0.17	0.05–0.29
					multifocality	OR _BMI_ =1.41	1.16–1.70
					ETE	OR _BMI_ =1.70	1.39–2.07
					LNM	OR _BMI_ =1.18	1.07–1.30
Kim et al.[51]	2013	RCS	2,057	Obesity	tumor size ≥ 1 cm	OR _BMI_ =1.41	1.10–1.81
					ETE	OR _BMI_ =1.88	1.06–3.32
					LNM	OR _BMI_ =1.46	0.78–2.74
					recurrence	p = 0.46^a^	NA
Warakomski et al.[50]	2018	RCS	177	Obesity	TNM stage	OR _WC_=6.35	1.78–22.69
Wu et al.[52]	2020	1:2 matched analysis	57	Obesity	recurrence	HR = 1.98	1.04–3.77
Feng et al.[53]	2019	RCS	417	Obesity	recurrence	p = 0.77^b^	NA
Hu et al.[54]	2019	RCS	649	FSG	tumor size ≥ 1 cm	OR = 2.60	1.18–5.7
Mele et al.[55]	2020	RCS	30	Insulin	recurrence	OR = 1.26	1.01–1.58
				HOMA-IR		OR = 1.71	1.02–5.14
Chen et al.[56]	2013	RCS	1,687	Diabetes	tumor size	HR = 1.12	0.99–1.28
					multifocality	HR = 0.22	0.04–1.10
					TNM stage	HR = 2.23	1.00-4.97
					DSS	p < 0.05	
					PFS	p < 0.05	
Li et al.[58]	2021	RCS	7,743	High TG	ETE	OR = 1.35	1.13–1.61
Zhou et al.[60]	2017	meta-analysis	24,655	High HDL-C	OS	HR = 0.82	0.75–0.90
					DFS	HR = 0.92	0.85–1.00

*PCS* prospective cohort study, *RCS* retrospective cohort study, *Met S* metabolic syndrome, *OR* odd ratio, *HR* hazard ratio, *CI* confidence interval, *LNM* lymph node metastasis, *TNM* tumor node metastasis, *ETE* extrathyroidal extension, *NA* not available, *BMI* body mass index, *WC* waist circumference, *TG* triglyceride, *HDL-C* high-density lipoprotein cholesterol, *FSG* fasting serum glucose, *HOMA-IR* homeostatic model assessment for insulin resistance, *DSS* disease free survival, *PFS* progression free survival, *OS* overall survival

^a^A result was gotten by Fisher’s exact test

^b^The researchers calculated the p value using univariate analysis

## Mechanism of the relationship between metabolic syndrome and thyroid Cancer

### Pathogenetic hypothesis

The current high incidence of Met S is accompanied by an increase in cancer cases worldwide. Whether there is a potential relationship between the two diseases, and if so, the mechanism of such a relationship needs to be futher explored. First, Met S may be an alternative marker for other cancer risk factors, such as reduced physical activity, high-calorie food intake, high dietary fat intake, low fiber intake, and oxidative stress. A healthy weight, regular physical activity, and a healthy diet can greatly reduce the lifetime risk of cancer and also affect the overall curative outcome and survival rate [62]. Second, the tumor may be the result of the long-term progression of Met S, as clinical evidence suggests that Met S is a risk factor for various cancers [8]. Finally, there may be shared a biology in the development of the two separate disease entities. For example, vascular endothelial injury can lead to the conducive increase of vascular endothelial growth factor (VEGF) for tumor angiogenesis.[63]

**Fig. 1:**
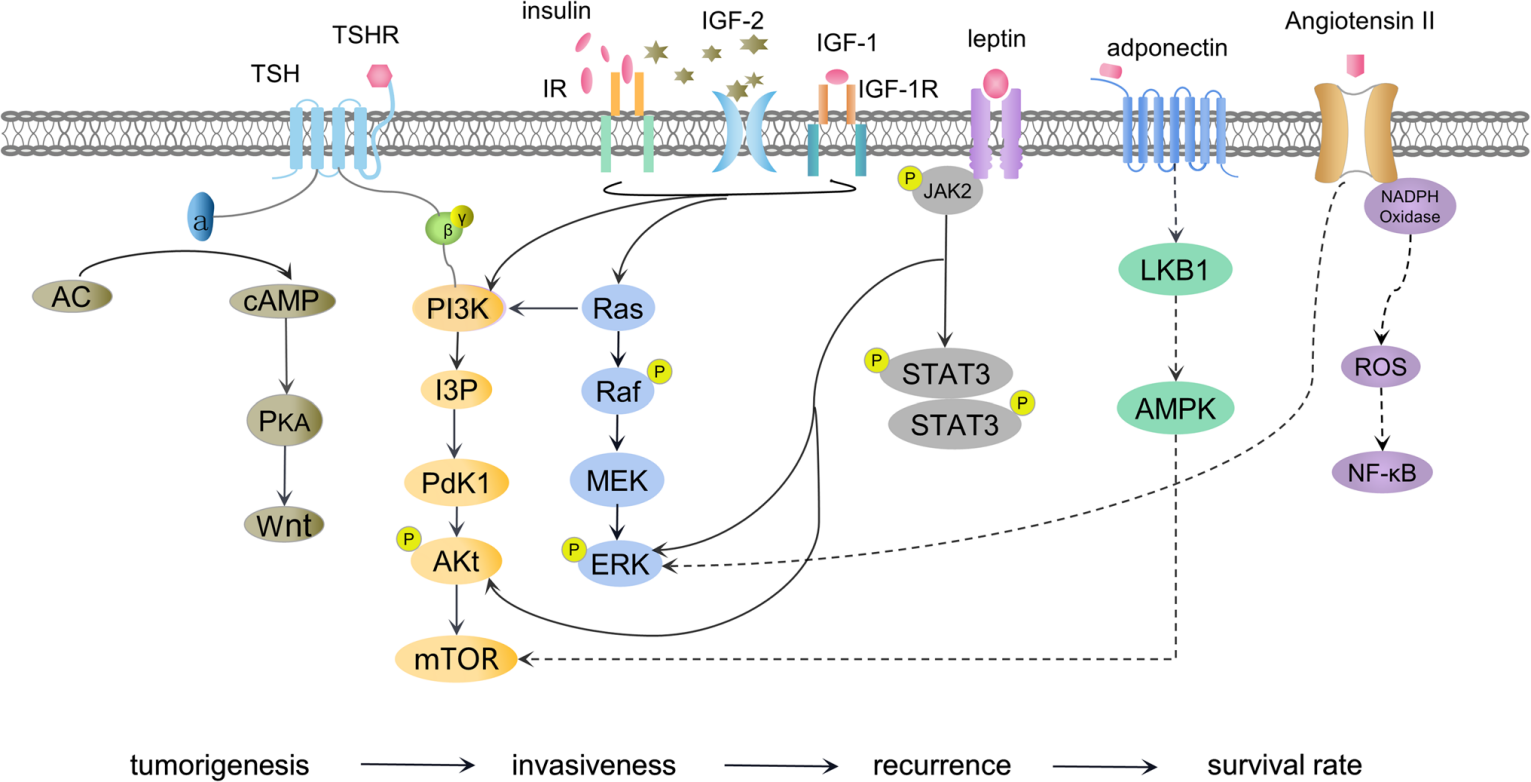
Mechanistic network of metabolic syndrome and thyroid cancer. *TSH* thyroid stimulating hormone, *TSHR* thyroid stimulating hormone receptor, *IR* insulin receptor, *IGF* insulin-like growth factor

Undoubtedly, as metabolic changes occur in vivo, disorders occur at the related molecular level. These molecules lead to the proliferation, invasion, and metastasis of TC through various signaling pathways and target genes (Fig. 1). Thyroid hormones are involved in the body’s energy balance, regulate glucose and lipid metabolism, and stabilize blood pressure. Higher hormone levels pose an increased risk of Met S [48]. Meanwhile, rich immune cells, epithelial cells, and adipocytokines result in the formation of a favorable microenvironment for tumor growth in obese individuals [64]. Hyperglycemia, especially type 2 diabetes, is often accompanied by insulin resistance, which in turn enhances the systemic effects of insulin-like growth factor (IGF). Moreover, the renin-angiotensin-aldosterone system (RAAS) is a major pathway for regulating blood pressure where the rise of Ang II can accelerate tumor progression. Additionally, lipids play an important role in cell membrane integrity and act as barriers for molecules entering and leaving cells. Cholesterol is a precursor of steroid hormones and tumor growth requires adequate cholesterol for the cell cycle and cell membrane formation.

### Thyroid-stimulating hormone (TSH)

A higher TSH level, even within the normal range, is currently considered as an independent risk factor for TC [65]. TSH acts on thyroid epithelial cells, which not only affects thyroid function but also stimulates cell proliferation. TSH is correlated with each Met S component even in normal thyroid status [66, 67]. This is demonstrated by the fact that the number of diabetes patients with elevated TSH levels is 3.5 times than that of non-diabetic patients [68]. TSH receptor is decreased in the adipose tissue of obese patients, and then the TSH level presents a compensatory increase [69]. TSH makes it easier for dividing epithelial cells to enter the G1 phase, maintain a high level of cAMP, and activate AC/cAMP/PKA pathway. Furthermore, cAMP can form a “signal network” of thyroid tumorigenesis by linking with the RAS/MAPK/ERK, PI3K/AKT, and Wnt pathways (Fig. 1). Thus, thyroid cells evolve into different tumor types and perform different biological behaviors [70, 71]. DTC cells retain their reactions to TSH, and TSH endocrine suppressive therapy is often used to control tumor progression after surgery. As described in the previous study [16], most TC cases related to Met S originated from thyroid epithelial cancer. Therefore, TSH may play a priming role in the development of Met S and TC.

### Insulin resistance

IGF axis may be another key pathway for inducing TC. When glucose intake and utilization are weakened, the body produces a large amount of compensatory insulin. This is known as insulin resistance, which is often accompanied by hyperinsulinemia and hyperglycemia. Insulin and IGF-1 are similar in structure and work together with TSH to promote DNA synthesis and cell proliferation. Meanwhile, insulin may inhibit the synthesis of IGF-binding proteins 1 and 2 (IGF-1 and IGF-2, respectively) and may promote the production of IGF-1 in the liver [72]. There is powerful evidence that cancerous thyroid cells often overexpress insulin receptors (IRs) and the IGF-1 receptor (IGF-1R) [71, 73]. IR exists in two subtypes: namely IR-A and IR-B. IR-A exists mainly in the fetus, and has an affinity with insulin and IGF-2, while IR-B binds only to insulin. Overexpression of IR may occur in the early stage of thyroid tumor formation and is key to the over-activation of the IGF system [73]. The two isoforms can hybridize with IGF-1R to form a hybrid IR/IGF-1R. This hybrid receptor binds to IGF-1 and further expands its action site. Cancerous thyroid cells can produce IGF-1 and IGF-2 locally: stromal cells secrete IGF-1, while IGF-2 is produced by tumor cells [73]. That IGF-2 regulates the IR-A excessive activation ultimately influences TC invasiveness, stem celllike characteristics, and response to treatment [71, 74, 75]. Thus, inhibiting IGF-2/IR-A may become a new idea for targeted therapy [76, 77].

In addition, hyperglycemia provides sufficient energy for rapid tumor growth, while damaging mitochondrial function, enhancing oxidative stress, and aggravating DNA damage [78, 79]. Under high-glucose condition, cytoskeleton remodeling creates a good microenvironment and facilitates cell metastasis and epithelial-mesenchymal transformation [79]. Therefore, insulin resistance is the central part of Met S and enhances the effects of the TSH and IGF systems. Repairing insulin resistance condition can inhibit tumor growth and proliferation, ameliorate the curative outcome of treatment, and uplift patients’ prognosis.

### Adipocytokines

Fat accumulation induces insulin resistance and long-term chronic inflammation, thereby breaking the stability of systemic metabolism. Leptin and adiponectin are the main hormones secreted by adipose tissue. The former binds receptors to suppress appetite and speed up energy metabolism. The latter is a protective factor with anti-inflammatory, insulin sensitization, and anti-arteriosclerosis actions. Obese people are prone to elevated leptin levels and decreased adiponectin levels [80]. Meanwhile, the common fat cells around the tumor could undergo morphological and functional changes. These tumor-associated adipocytes are often located at the interlaced sites of the tumors and the surrounding fat. The neck is a common site for fat accumulation, and lymph nodes are closely connected to adipocytes. TC patients are more likely to suffer from lymph node metastasis, so the role of tumor-related adipocytes cannot be ignored. These cells can gradually exhibit delipidation and dedifferentiation, and can gradually become severe endocrine entities. They may start to secret adipokines and inflammatory factors, the most important of which are leptin, adiponectin, and Visfatin [81].

#### Leptin

Siemińska et al. found that leptin levels were positively correlated with abnormal metabolic components in postmenopausal women [82]. Studies have shown that leptin plays an important role in dysmetabolic conditions, immunological functions, and tumor progression [83]. The leptin levels of PTC patients increased before surgery and decreased after total thyroidectomy, but were still higher than those in the healthy controls [84]. Eighty percent of PTC overexpress leptin receptors known as long and short isoforms [85, 86]. Leptin binds its long receptor to increase cell proliferation and inhibit the apoptosis by activating PI3K/AKT pathway and phosphorylating JAK2. (Fig. 1) [85, 86]. In addition, leptin induces the phosphorylation of ERK and then enhances the migration of malignant cells [87]. Studies have shown that leptin and its receptors are associated with the aggressiveness of DTC and that patients with high leptin have worse short prognoses [88].

#### Adiponectin

Cheng et al. found that TC patients have lower levels of circulating adiponectin than the healthy population, and that the adiponectin receptor is expressed more highly in cancer tissues than that in normal thyroid tissues [89]. Adiponectin working together with receptors can upregulate the LBK1 and AMPK levels [90]. AMPK activation is a crosstalk for adiponectin to exert its antitumor properties. Because it strengthens the body’s defense mechanism, but also inhibits the carcinogenic signal pathway. AMPK directly upregulates tumor suppressor genes (p27, p21, and p53) and prevents cell miosis and apoptosis via PP2A [91-93]. Cyclin D1 and mTOR, important cell proliferators to control cell cycles, are downregulated by AMPK [91, 94]. In addition, eEF2 kinase is activated by AMPK to induce cytotoxic autophagy and to reduce VEGF output [91, 95]. This makes tumors difficult to protein formation and angiogenesis. Nigro et al. [96] showed that adiponectin treatment can inhibit PTC growth and proliferation in a time- and dose-dependent manner. However, further verification is still needed in TC, even if adiponectin has been proven to play a protective role in multiple malignancies through the aforementioned mechanisms.

#### Visfatin

Visfatin is a rate-limiting enzyme for ATP production that catalyzes the synthesis of nicotinamide adenine dinucleotide (NAD+). Visfatin is mainly produced by visceral fat and has been demonstrated to promote cell growth in a variety of tumors [97]. Visfatin can be involved in the glycolysis, DNA repair, and angiogenesis of tumor cells through NAD+. Compared with benign tissue, visfatin is largely secreted in well-differentiated thyroid carcinomas and is positively correlated with later tumor stages [98, 99]. Studies have shown that common PAX8/PPAR rearrangement, PTEN, and PI3K gene mutations upregulate the levels of HIF-1 and STAT3, which jointly promote the visfatin of TC in abundance [98]. STAT3 also adjusts the release of interleukin-6 (IL-6) and further exacerbates the inflammatory response.

Leptin, adiponectin, and visfatin indirectly participate in the tumor formation process through key molecules such as AMPK, STAT, and PI3K. They also improve insulin resistance and assist in secreting related inflammatory molecules. Adipokines may play a supporting role in the relationship between Met S and TC. However, more clinical evidence and basic experiments are still needed to verify their biological pathogenesis.

### Angiotensin II

RAAS known for its role in BP regulation also play a considerable part in cancer progression. Angiotensin-converting enzymes (ACE and ACE2) regulates RAAS balance through producing respectively Ang II, a powerful vasoconstrictor and cell proliferator, and its antagonist Ang - (1-7). Notably, the ACE2 levels were lower in samples with larger thyroid tumors and distant metastases [100]. Ang II is a commonly active peptide involved in blood pressure regulation and tumor formation. It has opposite effects upon binding with Ang II receptor 1 (AT1) and Ang II receptor 2, whereas only AT1 has been confirmed to be expressed in the thyroids of mice [88]. After binding to AT1, Ang II is directly activated to promote mitosis and induce cell proliferation through MAPK/STAT3 and PI3K/AKT pathways [101, 102]. Furthermore, AT1 interacts with nicotinamide adenine dinucleotide phosphate oxidase to produce a large number of reactive oxygen species, activate NF- κB, and inhibit apoptosis [101]. Activated AT1 participates in tumor angiogenesis by facilitating the expression of matrix metalloproteinases, VEGF, and its receptors [101, 102]. Studies have shown that VEGF is overexpressed in PTC and is related to lymph node metastasis [103]. The fact that the tumor size shrunk after treatment with AT1 antagonists supports the role of AT1 in tumor formation [104]. In addition, RAAS reshapes the extracellular matrix and establishes an inflammatory microenvironment conducive to tumor survival [102]. Ang II finally takes part in distant metastasis by regulating adhesion, migration, and invasion [105].

### Others

The relationship between Met S and TC may also involve etiology such as inflammation, estrogen, and physical factors. Each Met S component could induce systemic chronic inflammation, produce reactive oxygen species, and cause cell damage [106]. A meta-analysis showed that the levels of inflammatory factor, such as TNF - α and IL-6, were significantly higher in TC patients than in healthy people [107]. Second, estrogen stimulates growth but inhibits the differentiation of thyroid stem cells [108]. It combines with receptor-β to counter swelling, whereas with receptor-α to promote cell growth and proliferation. Increased receptor-α levels in postmenopausal women may indicate their poor TC prognosis [109]. In addition, 27 hydroxycholesterols (27 HC), products of cholesterol metabolism, could interact with estrogen receptors and activate EGFR/AKT and EGFR/ERK signaling pathways. These events increase tumor angiogenesis and restrain apoptosis [110]. Revilla et al. showed that 27-HC increased and LDL-C decreased in patients with invasive TC, but there was no significant difference in HDL-C levels between these patients and those with well-differentiated TC and benign thyroid tumors [111]. However, as only 89 patients were included in this study, the role of cholesterol in TC needs to be further explored. Finally, thick adipose tissue delays the detection of TC through palpation, which increases surgical difficulty and length and ultimately leads to a poor prognosis.

## Conclusion

To date, there is insufficient research on the relationship between Met S and TC risk and prognosis. Most studies suggested that Met S components increased the patient’s risk of TC with gender differences. There is a correlation between these metabolic factors and PTC aggressiveness, but the prognostic relationship is unclear. Abnormal metabolism keeps the body in a state of inflammation for a long time. TSH may trigger neoplastic transformation. Insulin resistance is the core component, supplemented by adipokines, Ang II, and estrogen. All of them together drive the development of TC. cAMP, IGF axis, Ang II, and AMPK-related signaling pathways may give new targets for TC therapy. Indicators directly reflecting the metabolism are expected to become new diagnostic markers. Hence, weight management and a healthy diet can perfect body metabolism, reduce TC risk, and ameliorate prognosis.
